# Characterization of Ti/SnO_2_ Interface by X-ray Photoelectron Spectroscopy

**DOI:** 10.3390/nano12020202

**Published:** 2022-01-08

**Authors:** Miranda Martinez, Anil R. Chourasia

**Affiliations:** Department of Physics & Astronomy, Texas A&M University-Commerce, Commerce, TX 75429, USA; mmartinez72@leomail.tamuc.edu

**Keywords:** tin, titanium, tin oxide, X-ray photoelectron spectroscopy

## Abstract

The Ti/SnO_2_ interface has been investigated in situ via the technique of x-ray photoelectron spectroscopy. Thin films (in the range from 0.3 to 1.1 nm) of titanium were deposited on SnO_2_ substrates via the e-beam technique. The deposition was carried out at two different substrate temperatures, namely room temperature and 200 °C. The photoelectron spectra of tin and titanium in the samples were found to exhibit significant differences upon comparison with the corresponding elemental and the oxide spectra. These changes result from chemical interaction between SnO_2_ and the titanium overlayer at the interface. The SnO_2_ was observed to be reduced to elemental tin while the titanium overlayer was observed to become oxidized. Complete reduction of SnO_2_ to elemental tin did not occur even for the lowest thickness of the titanium overlayer. The interfaces in both the types of the samples were observed to consist of elemental Sn, SnO_2_, elemental titanium, TiO_2_, and Ti-suboxide. The relative percentages of the constituents at the interface have been estimated by curve fitting the spectral data with the corresponding elemental and the oxide spectra. In the 200 °C samples, thermal diffusion of the titanium overlayer was observed. This resulted in the complete oxidation of the titanium overlayer to TiO_2_ upto a thickness of 0.9 nm of the overlayer. Elemental titanium resulting from the unreacted overlayer was observed to be more in the room temperature samples. The room temperature samples showed variation around 20% for the Ti-suboxide while an increasing trend was observed in the 200 °C samples.

## 1. Introduction

Tin oxide (SnO_2_) has attracted significant attention due to its interesting properties. It is proving to be a possible alternative to TiO_2_ as an electron transporting layer in perovskite solar cells. The oxide has a lower crystallization temperature than that of TiO_2_. The TiO_2_ film requires a high annealing temperature (>450 °C) to obtain high quality films. This increases both the possibility of chemical interaction with the underlying substrates and interdiffusion [1,2]. The lower formation temperature makes it easier to crystallize and dope the tin oxide. Many materials as dopants (such as Mg, Li, Al, and Sb) to SnO_2_ have been investigated [3,4,5,6]. Tin oxide therefore becomes suitable for use in flexible solar cells, tandem solar cells, and large-scale commercialization at a lower cost [7,8,9]. It has been found to have excellent chemical stability, UV-resistance, and good antireflection as compared to TiO_2_. For example, Yahi et al. [10] have reported the average transmittance of SnO_2_ films to be around 80%. These properties increase the stability and lifespan of the devices [11].

The tin oxide and titanium oxide (TiO_2_) have found applications as gas sensors [12,13,14,15,16], photocatalysts [17,18,19,20,21], photovoltaic [22,23,24], optoelectronics [25,26,27,28], etc. Nanocomposites of these two oxides have found use in technological applications in various fields [29,30,31,32,33,34,35,36]. To increase the photocatalytic activity of photocatalysts, creation of nanocomposite of these two oxides (SnO_2_ and TiO_2_) represents a promising method. These two oxides bear some resemblance in terms of their material properties such as their structure, wide band gap, high transmittance, multi-valency state, and high melting point. SnO_2_ plays an essential role in nanocomposite structures with TiO_2_ due to the production of more hydroxyl radicals in such a composite compared to other oxides such as ZnO, WO_3_, and Fe_2_O_3_ [37]. A wide range of properties that could be obtained in the TiO_2_-SnO_2_ nanocomposites depend upon their synthesis method and precursor type. Several studies have been focused on the creation of TiO_2_-SnO_2_ nanocomposites in order to increase their photoactivity. Various methods used for the creation of the nanocomposites include sol-gel [38,39,40,41], hydrothermal synthesis [42,43], chemical vapor deposition [44], spray and laser pyrolysis [45,46,47], coprecipitation [48,49], and green [50]. The significant differences that exist between the present investigation and those employed by other researchers lie primarily in the method of preparing the Ti/SnO_2_ interface. In the present investigation, thin films of tin and titanium were deposited by the technique of electron-beam evaporation. Since the depositions have been carried out under high vacuum, such a method of preparation provides a contaminant free specimen.

In the present investigation, the chemical reactivity at the titanium-stannic oxide (Ti/SnO_2_) interface has been investigated as a function of the thickness of the titanium overlayer and the substrate temperature. Thin films of titanium were deposited onto the stannic oxide substrate. The deposition of the titanium overlayer was performed under two different processing conditions: the substrate kept at ambient temperature and at 200 °C during the deposition. The 200 °C temperature was chosen in order to view significant changes at the interface. The interfaces have been characterized in situ by the technique of XPS. This technique is sensitive to the changes in the chemical states and thus becomes suitable for this investigation [51]. The results have been compared with the spectral data from elemental and oxidized tin and titanium. The investigation shows considerable reactivity at the interface with the formation of the oxides of titanium followed by the reduction of stannic oxide to elemental tin. The nature of the constituents at the interface was observed to depend upon the processing conditions. The amount and nature of constituents at any interface depend upon the following factors: annealing temperature, annealing time, and the thickness of the overlayer. The present investigation points to investigating the interface under a fixed processing condition as required for a particular application. Nevertheless, the results of this investigation provide an insight in preparing the controlled thicknesses of the nano-structured Ti/SnO_2_ interface.

## 2. Experimental

The XPS investigation was performed by using the Physical Electronics PHI 5100 ESCA system (Chanhassen, MN, USA). The magnesium anode (energy = 1253.6 eV) was used as the source of excitation. For calibration purposes, pure silver, gold, and copper samples were used. The Cu 2p_3/2_ an Au 4f_7/2_ peaks were set to give a binding energy (BE) difference of 848.6 eV. This established the linearity of the BE scale. The Ag 3d_5/2_ core level peak was set at 368.2 eV. The full width at half maximum of the Ag 3d_5/2_ peak was determined to be 1.8 eV which gives a measure of the resolution. In this investigation, the high resolution spectra were taken with a pass energy of 35.75 eV.

A deposition chamber has been attached to the XPS system. The sample can be transferred between these two chambers, thereby allowing in situ characterization of the samples. An Oxford Applied Research electron gun (model EGN4) mounted in the deposition chamber was used for the deposition of the samples. Four samples in the form of wire (or in crucibles) can be mounted onto this gun. The sample to be deposited can be chosen by selecting the appropriate filament. The base pressure in both the deposition chamber and the XPS chamber was better than 2 × 10^−9^ Torr and rose to about 9 × 10^−9^ Torr during the deposition. Pure elements of tin and titanium (of purity 99.999%, Alfa Aesar, Haverhill, MA, USA) have been used in the present investigation. The deposition chamber is also equipped with a quartz crystal monitor. This oscillator has been used to calibrate the rate of deposition of the elements used in this investigation. For this purpose, a thickness controller unit (OMNI III, Phelps Electronics, Inc., Oxford, NJ, USA) was employed. The emission current in the EGN4 was set to some value and the time for deposition of a fixed thickness of the material was monitored. The emission current was changed to some other value and the experiment repeated. From these data, a plot of the emission current versus the rate of evaporation was generated. A straight line fit to the plotted data provided the calibration for the thickness of the material deposited.

The tin oxide (SnO_2_) on the silicon substrate was formed by the following procedure. The silicon substrates were cleaned with a dilute HF acid (Thermo Fisher Scientific Chemicals, Inc., Ward Hill, MA, USA) for 30 s and mounted into the deposition chamber. About 30 nm of elemental tin deposited on this Si substrate. Following this deposition, the sample was removed from the processing chamber and was oxidized in a quartz tube furnace under a constant flow of pure oxygen. During the oxidation of tin, the substrate temperature was maintained at 300 °C for 0.5 h. After this time the flow of oxygen was cut off and the sample was allowed to cool down to the room temperature at the natural rate. The sample was then loaded into the characterization chamber and the x-ray photoelectron spectrum in the 3d region of tin was recorded. The formation of the SnO_2_ phase of tin oxide was confirmed by comparing the shape and the BE positions of the peaks with those reported earlier [52]. After confirming the formation of SnO_2_, the sample was transferred into the deposition chamber where the deposition of thin films of titanium was carried out.

Different samples were prepared by depositing varying thicknesses of the titanium overlayer (0.3, 0.5, 0.7, 0.9, and 1.1 nm) on the SnO_2_ substrate. Two sets of samples were prepared. In one set, the titanium overlayer was deposited on the SnO_2_ substrate kept at room temperature. In the other set, the substrate temperature was kept at 200 °C while the titanium overlayer was being deposited.

## 3. Results and Discussion

The Ti/SnO_2_ interface was characterized in situ by the technique of XPS. The tin 3d, titanium 2p, and oxygen 1s core levels were recorded in the two sets of the samples. Each of the spectra represents an average of thirty scans. Since the identification of the chemical states could be easily obtained from the tin and the titanium core level spectral data, the oxygen region has not been included in the discussion.

### 3.1. Room Temperature Deposition

#### 3.1.1. Sn 3d Region

**Figure 1:**
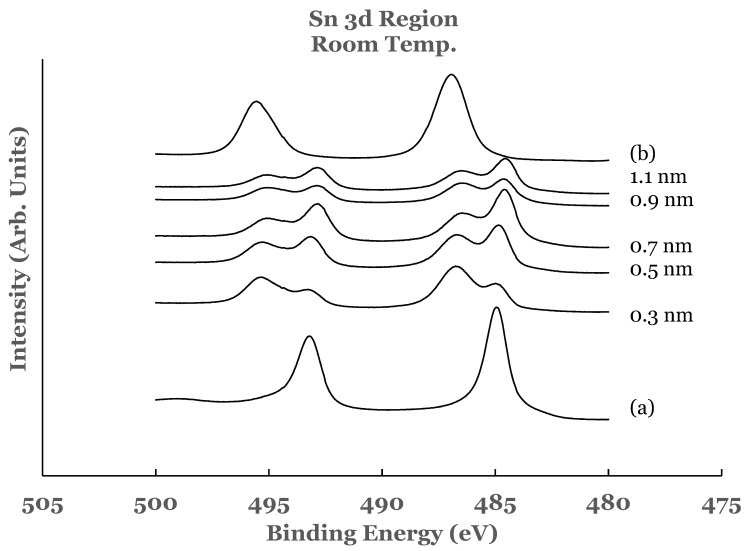
Spectral data in the Sn 3d region as a function of the thickness of the titanium overlayer. The overlayer was deposited at room temperature. Curve (a) corresponds to elemental Sn while curve (b) corresponds to SnO_2_.

In order to analyze the experimental data from the Ti/SnO_2_ interface, spectral data from elemental tin and tin oxide were recorded. For this purpose, a 20 nm thick film of tin was deposited on a silicon substrate at room temperature. The spectrum from this sample is shown as curve (a) in Figure 1. In this spectrum the 3d_5/2_ and 3d_3/2_ core level peaks are observed to be at BE values of 484.95 and 493.18 eV, respectively. These values are in agreement with those reported for elemental tin by other researchers [53,54]. The spectrum indicates that under the deposition conditions used in this investigation, tin gets deposited as elemental tin on silicon. The tin oxide (SnO_2_) was formed by oxidizing a similar sample of tin in a quartz tube furnace. The spectrum obtained from this sample is included as curve (b) in Figure 1. The core level peaks obtained from the SnO_2_ sample are seen to be shifted to the high BE side as compared to those for elemental tin. The BEs of the 3d_5/2_ and 3d_3/2_ core level peaks in this curve are measured to be 486.93 eV and 495.56 eV, respectively, and are in agreement with those reported by other researchers [53,54].

Figure 1 also includes the high resolution spectra in the 3D region of tin obtained from the Ti/SnO_2_ interfaces for the different thicknesses of the titanium overlayer. These spectra are normalized to have equal intensity at 482 eV. The normalization of the spectra facilitates comparison for increase or decrease in the intensity in the core level region. The spectrum from the 0.3 nm sample shows the presence of a shoulder on the low BE side of each of the tin oxide core level peaks. Upon comparison with the elemental spectrum, the presence of this shoulder is attributed to the presence of elemental tin at the Ti/SnO_2_ interface. The spectral features therefore indicate that the room temperature deposition of the 0.3 nm overlayer of titanium on SnO_2_ results in the partial reduction of the oxide into elemental tin. This is corroborated by the investigation of the 2p region of titanium in these samples, as explained later in this paper.

The spectral data from the 0.5, 0.7, 0.9, and 1.1 nm thick titanium overlayers are similar to those observed for the 0.3 nm sample. In all of these samples, there is no change in the shape of the spectral features. The intensity of the core level peaks corresponding to SnO_2_ is observed to decrease while that due to elemental Sn to increase as a function of the thickness of the titanium overlayer. Only a partial reduction of SnO_2_ is observed even with the lowest thickness of the titanium overlayer.

**Figure 2:**
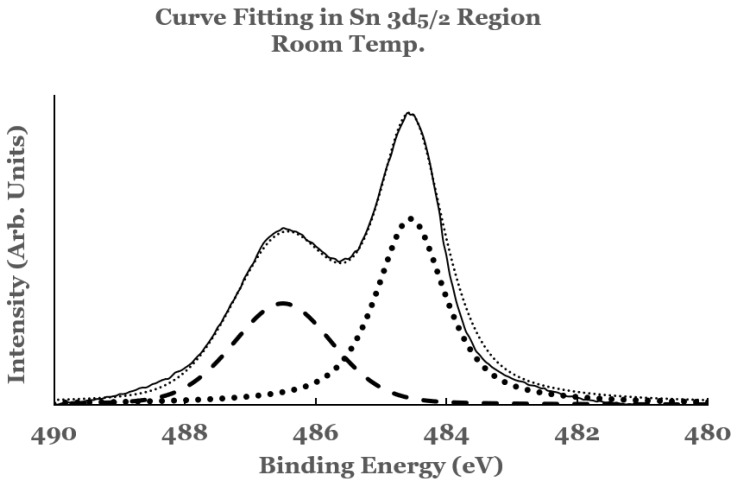
Curve fit for the 3d_5/2_ region of tin for the 1.1 nm titanium overlayer deposited at room temperature. The thin solid line represents the experimental data. The thick dotted line represents the spectrum from elemental Sn while the thick dashed line that from SnO_2_. The thin dotted line represents the superposition from elemental Sn and SnO_2_.

**Figure 3:**
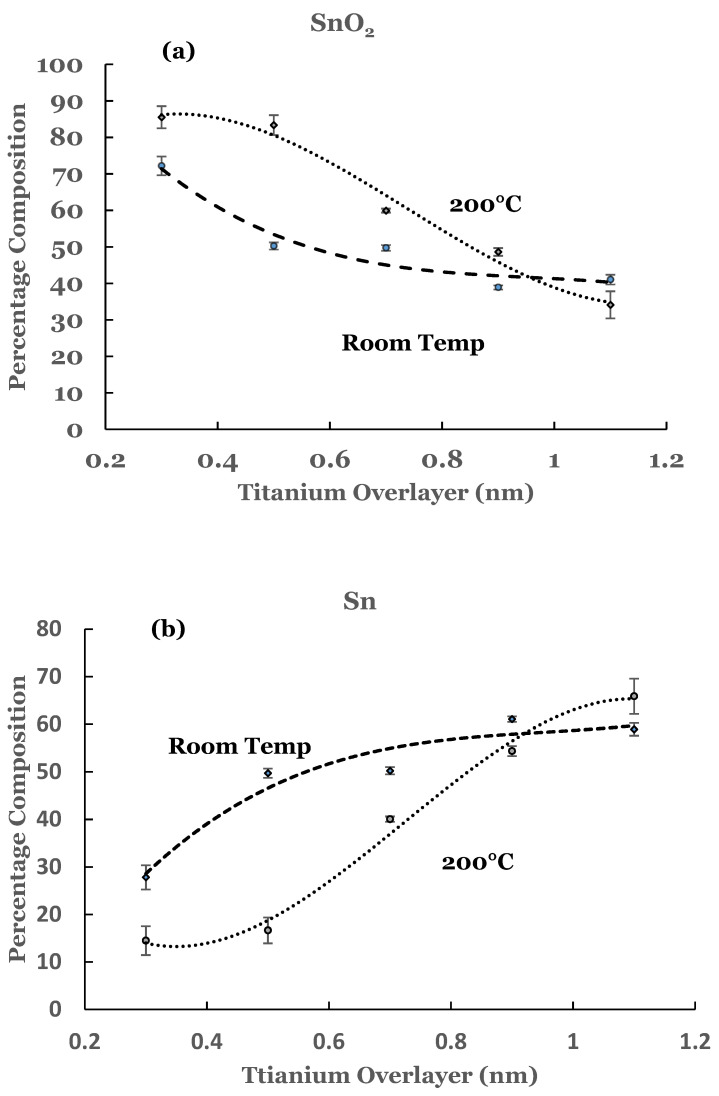
(**a**) Percentage composition of SnO_2_ as a function of the thickness of the titanium overlayer in the two sets of samples, and (**b**) percentage composition of elemental Sn as a function of the thickness of the titanium overlayer in the two sets of samples. The lines serve as a guide for the trend in the data. The error bars are also included.

To estimate the amounts of the SnO_2_ and elemental Sn present at the interface, a curve fit was carried out to the spectral data. An example of curve fit is given in Figure 2 for the 1.1 nm sample. A Shirley background was subtracted from the spectrum before the curve fit. The elemental and the oxide spectra were scaled to match the spectral data from the sample. The relative percentages of SnO_2_ and elemental Sn were determined from the areas under the corresponding fitting curves, normalized to the total area of the 3D peak. Plots of the percentage composition of SnO_2_ and elemental Sn thus determined as a function of the thickness of the titanium overlayer are shown in Figure 3a,b. The dashed lines serve as a guide to represent the trend in the data. From these figures it is observed that SnO_2_ shows a decreasing trend while elemental Sn shows an increasing trend with the increase in the titanium overlayer. The decrease in SnO_2_ is significant until 0.5 nm Ti overlayer and then it becomes gradual for thicknesses greater than this. This can be understood from the amount of titanium available to interact with SnO_2_ at the interface. Till 0.5 nm thickness, significant amount of titanium interacts with SnO_2_. As the thickness increases, the underlying layers prevent further interaction with the SnO_2_ substrate. The study therefore indicates the presence of SnO_2_ and reduced elemental tin at the Ti/SnO_2_ interface.

#### 3.1.2. Ti 2p Region

**Figure 4:**
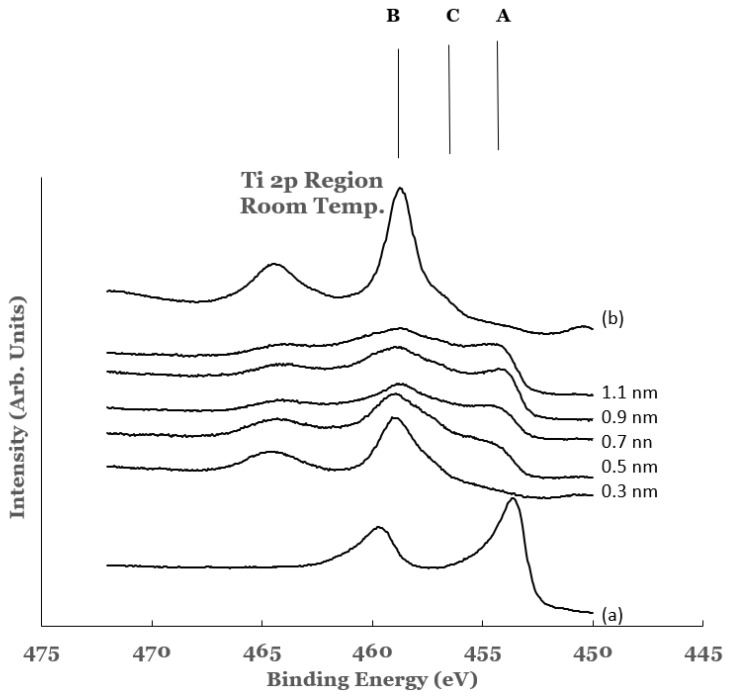
Spectral data in the Ti 2p region as a function of the titanium overlayer. The overlayer was deposited at room temperature. Vertical lines represent the positions of the 2p_3/2_ core level of titanium in the different chemical states of titanium. Line A represents the position of the 2p_3/2_ core level for elemental Ti, line B that for TiO_2_, and line C that for Ti-suboxide. Spectrum (a) corresponds to that for elemental Ti while spectrum (b) to that for TiO_2_.

The titanium 2p region in the samples investigated are shown in Figure 4. The spectra are normalized to have equal intensity at 450 eV. The data from elemental titanium and TiO_2_ are also included in this figure. The spectrum of elemental titanium was obtained by depositing about 20 nm of elemental titanium on a silicon substrate (Ti/Si). The spectrum from this sample is shown as curve (a) in Figure 4. The spectral features show BE values of 454.1 and 460.2 eV, respectively, for the 2p_3/2_ and 2p_1/2_ core level peaks. These values are in agreement with those reported in literature for elemental titanium [55,56]. The titanium oxide (TiO_2_) was formed by oxidizing the Ti/Si sample in an oxygen environment using a quartz tube furnace. The spectrum obtained from the oxide is included as curve (b) in Figure 4. The core level peaks in this spectrum are observed to be broader and shifted to the high BE side as compared to those for elemental titanium. The BEs of the 2p_3/2_ and 2p_1/2_ core level peaks are measured to be 458.9 and 464.7 eV, respectively. These values are in good agreement with those reported by other researchers for TiO_2_ [57,58].

The 2p region of titanium in all of the spectra from the Ti/SnO_2_ samples is observed to contain broad spectral features. The presence of these features indicates chemical reactivity at the Ti/SnO_2_ interface. The features can be divided into three regions: region A (centered around 453 eV), region B (centered around 458 eV), and region C (centered around 456 eV). The intensities in these regions are compared with those from elemental Ti and TiO_2_ to estimate the extent of the chemical reactivity. Region A corresponds to that from the 2p_3/2_ core level peak of elemental titanium. It represents the amount of elemental titanium left unreacted from the overlayer. It is observed to increase as a function of the thickness of the overlayer. Region B corresponds to that from the 2p_3/2_ core level peak of titanium in TiO_2_. It represents the amount of TiO_2_ formed at the interface as a result of chemical interaction between Ti and SnO_2_. In the 0.3 nm sample, larger intensity in region B and a very small intensity in region A are observed. The increase in the intensity in region A and the decrease in the region B for other samples are attributed to the increase and decrease of elemental Ti and TiO_2,_ respectively, at the interface. The spectral data therefore indicate intense chemical reaction at the Ti/SnO_2_ interface. The amount of unreacted titanium is observed to increase with the increase in the thickness of the Ti-overlayer. The extent of the chemical reactivity at the interface is observed to decrease with this increase.

**Figure 5:**
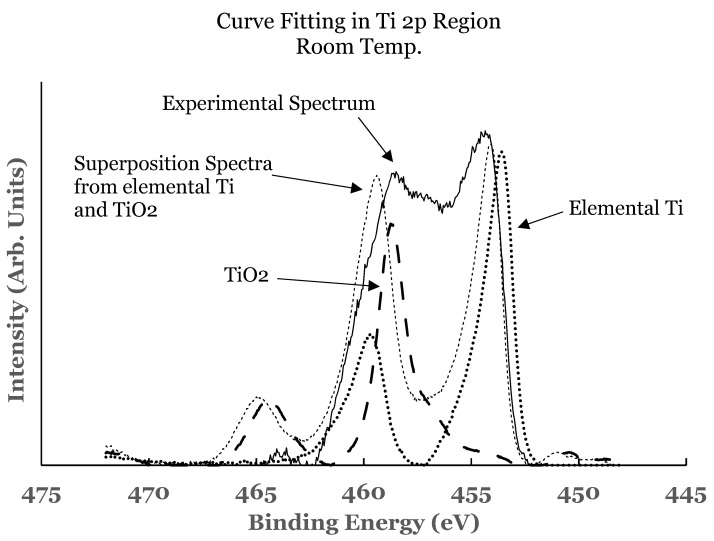
Curve fit for the 2p region of titanium for the 1.1 nm overlayer deposited at room temperature. The thin solid line represents the experimental data. The thick dotted line represents the spectrum from elemental Ti while the thick dashed line that from TiO_2_. The thin dotted line represents the superposition from elemental Ti and TiO_2_.

**Figure 6:**
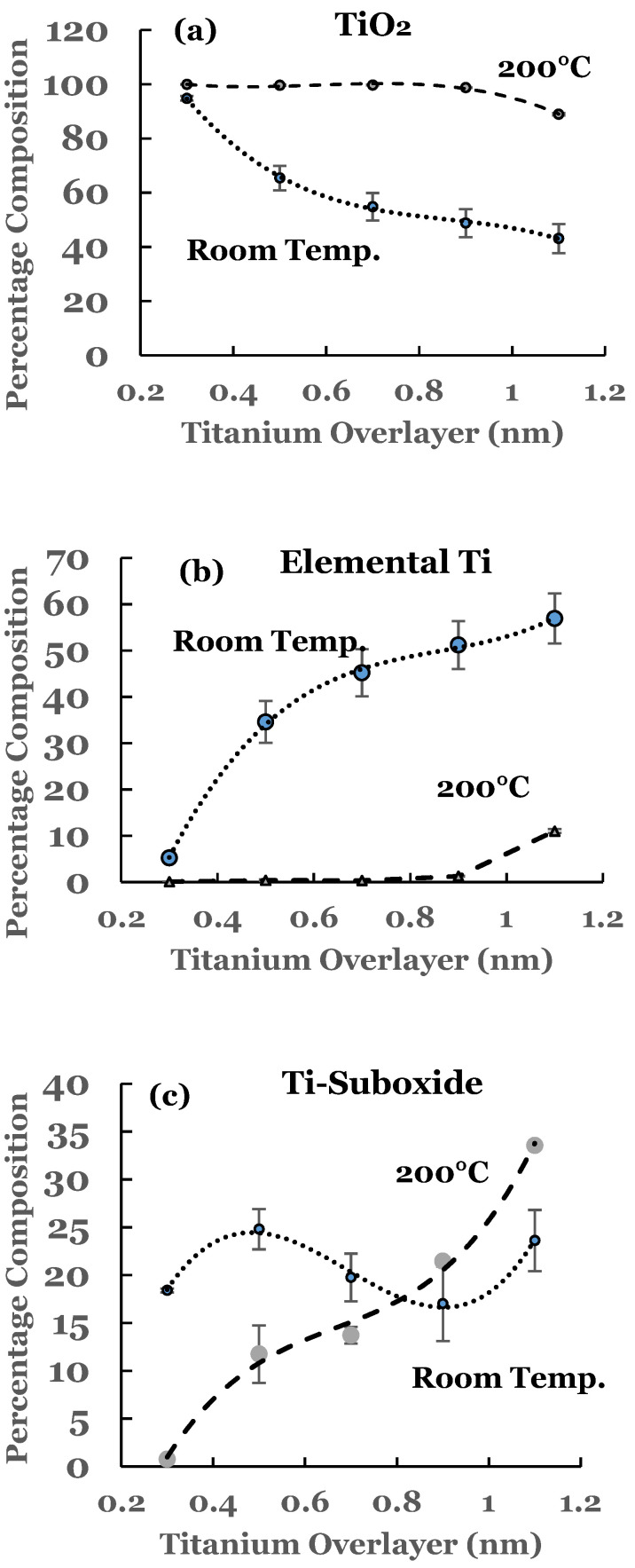
(**a**) Percentage composition of TiO_2_ as a function of the thickness of the titanium overlayer in the two sets of samples; (**b**) percentage composition of elemental Ti as a function of the thickness of the titanium overlayer in the two sets of samples; (**c**) percentage composition of Ti-suboxide as a function of the thickness of the titanium overlayer in the two sets of samples. The lines serve as a guide for the trend in the data. The error bars are also included.

A curve fitting was also performed in the 2p region to estimate the amounts of the constituents present at the interface. A Shirley background was subtracted from the experimental curve before the curve fit. The spectra from elemental Ti and TiO_2_ were superimposed on the experimental curve. For this, their intensities were scaled to match one end of the spectrum. An example of such a curve fitting is shown in Figure 5 for the 1.1 nm sample. Due to the asymmetric shape of the core level peaks of titanium, a complete match of the fitted curve with the experimental one was not possible. Any excess intensity of the fitted curve was subtracted from the total area before estimating the percentage concentration. It is evident from this figure that the region C consists of additional intensity. This intensity is due to the suboxide of titanium resulting from incomplete chemical interaction between titanium and SnO_2_ at the interface. The results therefore show that the interface consists of TiO_2_, elemental Ti, and Ti-suboxide. The amounts of TiO_2_, unreacted Ti, and suboxide of Ti have been estimated from the spectral data. The areas under the curves were determined using the trapezoidal method. The relative percentage of each of the constituents was determined from these areas, normalized to the total area of the spectrum. The variations in the percentage of TiO_2_, elemental Ti, and Ti-suboxide in these samples as a function of the thickness of the titanium overlayer are shown in Figure 6a–c. The lines in these figures represent the guides for the variations. The TiO_2_ shows an initial decrease followed by saturation beyond the 0.5 nm of the titanium overlayer. The Ti-suboxide and unreacted titanium are observed to increase initially followed by flattening off for the same thickness of the titanium overlayer. These trends are in accord with those observed for elemental Sn and SnO_2_.

Different researchers have employed different techniques of curve fitting to quantify the presence of the constituents in a specimen. Saric et al. [59] have investigated the oxidation of cobalt metal by low-energy oxygen bombardment at room temperature. They have estimated the concentration fraction of CoO and Co_3_O_4_ in the samples through curve fitting. The 2p_3/2_ core level of elemental cobalt was fitted with an intense asymmetric peak with two small additional peaks. In the oxidized samples, they observed that at least eight fitting components were required to obtain a good fit. Due to the possibility of the presence of other sub-oxides of cobalt and the overlap of the fitted peaks, their method may not fully account for the fraction concentration of the constituents. Hong et al. [60] have investigated the Fe/CuO interface. They have estimated the amount of unreacted iron and Fe_2_O_3_ at the interface by modeling the spectra from the elemental iron and pure Fe_2_O_3_. The fitted spectra were calculated with the constraint: x + y = thickness of the deposited overlayer with x being the thickness of the elemental iron and y that for Fe_2_O_3_. The values of x and y were varied to get a good fit with the experimental curve. These methods may work if there were no sub-oxides present. In the present investigation, scaling the intensities of the peaks is expected to account for the various components to scale accordingly. Hence, the method of curve fitting employed in the present investigation is suitable for the estimation of the suboxide at the interface.

### 3.2. Substrate Temperature (200 °C) Deposition

#### 3.2.1. Sn 3d Region

**Figure 7:**
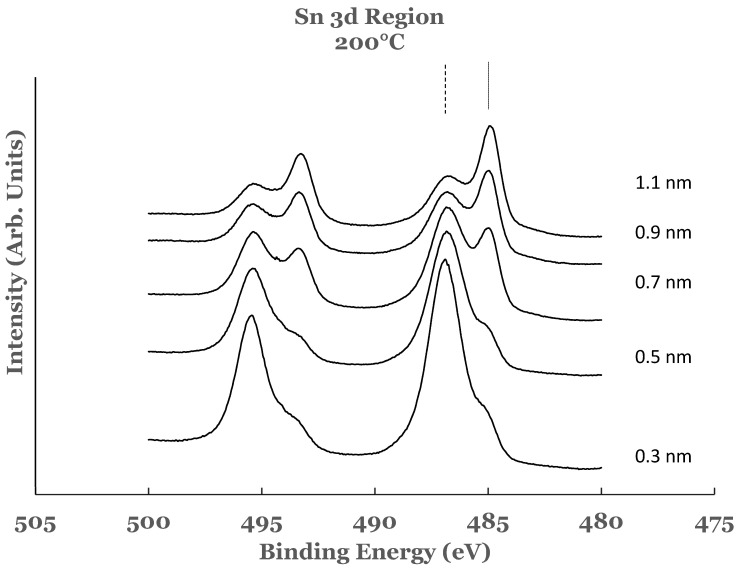
Spectral data in the Sn 3d region as a function of the titanium overlayer. The overlayer was deposited at the substrate temperature of 200 °C. The vertical lines represent the positions of the 3d_5/2_ core level. The solid line corresponds to elemental Sn while the dashed line to SnO_2_.

The high resolution spectra in the 3D region of tin obtained from the Ti/SnO_2_ interfaces for the titanium overlayer deposited at a substrate temperature of 200 °C are shown in Figure 7. These spectra are normalized to have equal intensity at 482 eV. The spectrum from the 0.3 nm sample shows the presence of shoulder on the low BE side of each of the tin oxide core level peaks. The presence of this shoulder is attributed to the reduction of SnO_2_ to elemental tin by the titanium overlayer. The intense peaks in this spectrum are due to the SnO_2_. The spectral features therefore indicate that the deposition of titanium at this substrate temperature results in the partial reduction of the oxide into elemental tin. The spectral data from the 0.5, 0.7, 0.9, and 1.1 nm thick titanium overlayers are similar to those observed for the 0.3 nm sample. In all of these samples there is no change in the shape of the spectral features. The intensity of the core level peaks corresponding to SnO_2_ is observed to decrease while that due to elemental Sn to increase as a function of the thickness of the titanium overlayer. As in the previous case, only a partial reduction of SnO_2_ is observed even with the lowest thickness of the titanium overlayer.

**Figure 8:**
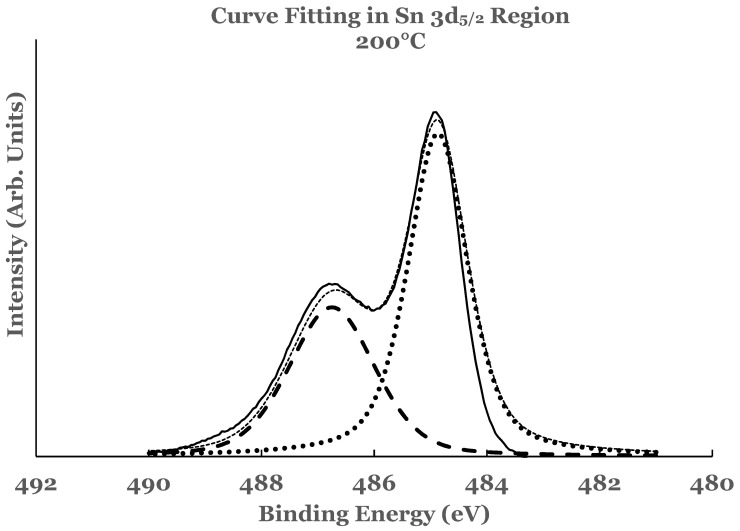
Curve fit for the 3d_5/2_ region of tin for the 1.1 nm titanium overlayer deposited at substrate temperature of 200 °C. The thin solid line represents the experimental data. The thick dotted line represents the spectrum from elemental Sn while the thick dashed line that from SnO_2_. The thin dotted line represents the superposition from elemental Sn and SnO_2_.

The amounts of SnO_2_ and elemental Sn at the interface were estimated for these sam-ples also. For this purpose a curve fit to the spectral data was performed as explained ear-lier. An example of curve fit is given in Figure 8 for the 1.1 nm of titanium on SnO_2_. The relative percentages of SnO_2_ and elemental Sn were determined from the areas of the cor-responding fitting curves, normalized to the total area of the 3d peak. Plots of the percent-age compositions of SnO_2_ and elemental Sn, thus determined as a function of the thickness of the titanium overlayer, are also included in Figure 3a,b. The dashed lines serve as a guide to represent the trend in the data. For these samples, a decreasing trend in SnO_2_ while an increasing trend in elemental Sn are observed with the increase in the titanium overlayer. Some differences exist when comparing these data with the room temperature data. The SnO_2_ concentration starts from a higher value in such samples and continues to show a decreasing trend for thicknesses greater than 0.9 nm. Similarly, the elemental Sn starts from a lower value and continues to show an increasing trend. The data from these samples therefore suggest that the titanium overlayer continues to interact with the underlying SnO_2_ layer for thicknesses greater than 0.9 nm. The increased interaction at the interface can be attributed to the thermal diffusion of the titanium overlayer into the underlying SnO_2_ in such samples. The study therefore indicates the presence of SnO_2_ and elemental Sn in greater amounts in such samples.

#### 3.2.2. Ti 2p Region

**Figure 9:**
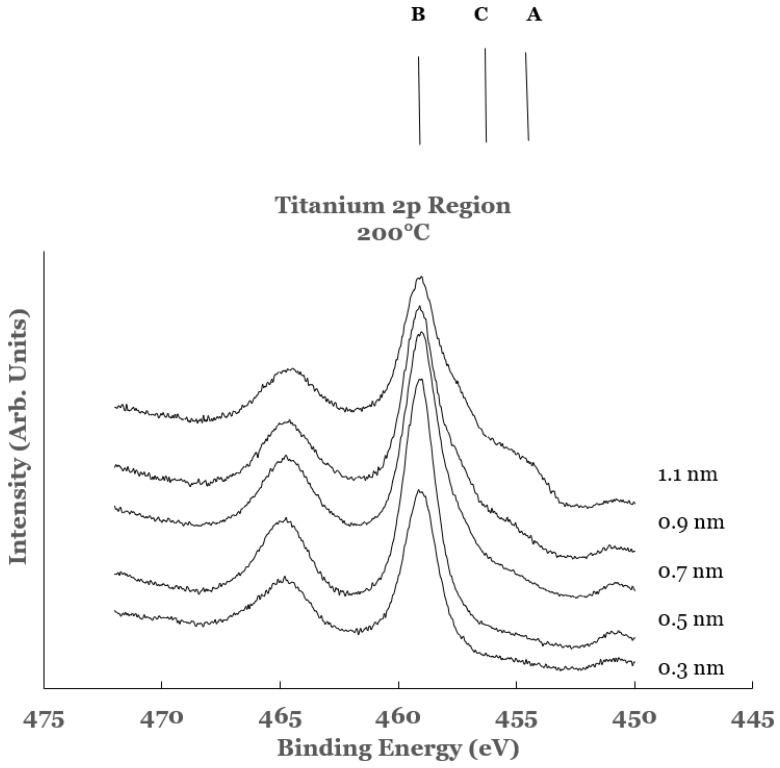
Spectral data in the Ti 2p region as a function of the titanium overlayer. The overlayer was deposited at the substrate temperature of 200 °C. Vertical lines represent the positions of the 2p_3/2_ core level of titanium in the different chemical states of titanium. Line A represents the position of the 2p_3/2_ core level for elemental Ti, line B that for TiO_2_, and line C that for Ti-suboxide.

The high resolution spectra in the 2p region of titanium obtained from the Ti/SnO_2_ interfaces for the titanium overlayer deposited at a substrate temperature of 200 °C are shown in Figure 9. The spectra are normalized to have equal intensity at 450 eV. The spectral features in these samples exhibit considerable chemical reactivity at the Ti/SnO_2_ interface. The intensity in the region A (centered around 453 eV and corresponds to the 2p_3/2_ core level of titanium in elemental titanium) is observed to increase for thicknesses greater than 0.5 nm. A large intensity in the region B (centered around 458 eV and corresponds to that from the 2p_3/2_ core level peak of titanium in TiO_2_) is observed in all of the samples. The intensity of this spectral feature is observed to decrease with increase in the overlayer thickness. The intensity in region C (centered around 456 eV and corresponds to that from the Ti-suboxide) is observed to increase for thicknesses greater than 0.5 nm. The spectral data therefore indicate intense chemical reaction at the Ti/SnO_2_ interface. The increase in the amount of unreacted titanium with the increase in the thickness of the Ti-overlayer represents a decrease in the extent of the chemical reactivity at the interface. A decrease in the intensity in region C is observed in these samples when compared with the room temperature data. This is interpreted as an increase in the TiO_2_ content in such samples resulting from the diffusion of the titanium overlayer into the underlying SnO_2_ layer.

**Figure 10:**
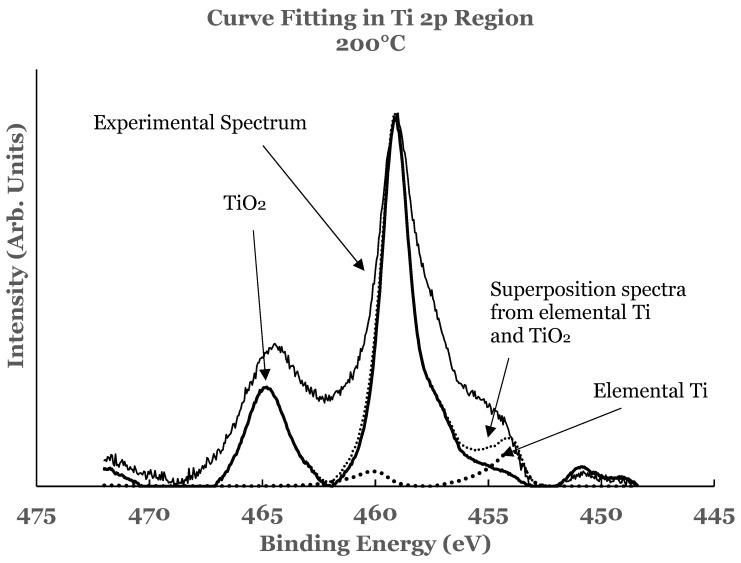
Curve fit for titanium for the 1.1 nm overlayer deposited at 200 °C substrate temperature. The thin solid line represents the experimental data. The thick dotted line represents the spectrum from elemental Ti while the thick solid line that from TiO_2_. The thin dotted line represents the superposition from elemental Ti and TiO_2_.

A curve fitting was also performed in the 2p region in these samples to estimate the amount of the constituents present at the interface. The details of curve fitting have been outlined earlier. An example of such a curve fitting is shown in Figure 10 for the 1.1 nm sample. It is evident from this figure that the region C consists of additional intensity. This intensity is due to the suboxide of titanium resulting from incomplete chemical interaction between titanium and SnO_2_ at the interface. The results therefore show that the interface consists of TiO_2_, elemental Ti, and Ti-suboxide. The amounts of TiO_2_, unreacted Ti, and suboxide of Ti have also been estimated from the spectral data of such samples.

The variations in the percentage of TiO_2_, elemental Ti, and Ti-suboxide in these samples as a function of the thickness of the titanium overlayer are also included in Figure 6a–c, respectively. The lines in these figures represent the guides for the variations. The percentage of TiO_2_ is observed to be around 100% till 0.9 nm thickness of the overlayer. This is followed by a decrease in its concentration beyond this thickness. The thermal diffusion of the Ti overlayer makes more titanium to be available for chemical interaction with SnO_2_. When compared with the room temperature data, the spectral data for these samples indicate complete oxidation of the titanium overlayer till 0.9 nm thickness. This variation is corroborated by the variation in the percentage for elemental Ti (Figure 6b). The elemental titanium is near zero and shows an increasing trend beyond the 0.9 nm thickness. The trend is markedly different from that of the room temperature data. The unreacted titanium shows a rather sharp increase in the percentage for the first two samples in the case of the room temperature deposition. This increase then becomes gradual for thicknesses beyond 0.5 nm. The variation in the Ti-suboxide is also included Figure 6c for these samples. Significant variation in the percentage is observed for thicknesses beyond 0.5 nm. The room temperature data, on the other hand, shows variation around 20% for all of the samples. The trends are in accord with those observed for elemental Sn and SnO_2_.

The interfacial reaction between Ti and SnO_2_ can be understood by considering the Gibbs free energy of the involved products. Gamsjager et al. [61] have compiled the Gibbs free energy data for SnO_2_ from various sources. The table A-58 in that reference lists a range of values between −367 and −372 kJ/mol. These values have been measured over a temperature range 673–1380 K. Schaefer has also compiled various values (Table 8 in Ref. [62]) from different researchers. For the present investigation, a value of −407 kJ/mol has been used for SnO_2_. Kim and Kang [63] have listed experimental values of the Gibbs free energy for various titanium oxides. The value of −659 kJ/mol has been used for TiO_2_ in the present calculation.

The spectral data for the room temperature Ti/SnO_2_ interface show the interfacial reaction to occur as:

Ti + SnO_2_ → TiO_2_ + Sn + SnO_2_ + Ti-suboxide (1)

Titanium exhibits oxidation states of +2, +3, and +4. The Ti-suboxide can therefore be attributed to the presence of Ti_2_O_3_ near the interface. The above reaction can then be written as:

5Ti + 4SnO_2_ → TiO_2_ + 4Sn + 2Ti_2_O_3_ (2)

Considering the above mentioned Gibbs free energy for the involved constituents (the value for Ti_2_O_3_ is −1430 kJ/mol, [64]), the Reaction (2) results in an approximate value for the change in Gibbs free energy as −1891 kJ/mol. This indicates that such an interfacial reaction can proceed thermodynamically.

For the deposition at the substrate temperature of 200 °C, the reaction occurs according to the following equation:

Ti + SnO_2_ → TiO_2_ + Sn (3)

which is also favored thermodynamically (change in Gibbs free energy is approximately −742 kJ/mol). The data also suggest that TiO_2_ is more readily formed at the interface when the substrate temperature is increased.

The partial reduction of SnO_2_ to SnO can also occur according to:

2Ti + 3SnO_2_ → 2TiO_2_ + 2SnO (4)

The value of the Gibbs free energy for the formation of SnO is −253 kJ/mol [65]. This yields a value of −1893 kJ/mol for the above reaction. However, the spectral data indicate that such a reaction is not favorable under the experimental conditions.

The analysis of the data thus provides a picture of the contents at the interface. The oxidized titanium and the reduced tin are present at the interface. As the titanium overlayer increases, the interfacial TiO_2_ layer serves as a barrier for further oxidation of titanium. Due to less availability of oxygen away from the interface, the subsequent titanium overlayer gets partially oxidized. This occurs until after a certain thickness the titanium overlayer results in the unreacted titanium. The present investigation points to the need for more experiments to establish a correlation between the amount of constituents and the substrate temperatures. The electrical properties of these interfaces can then be explored against particular device applications.

## 4. Conclusions

In conclusion, the Ti/SnO_2_ interfaces have been investigated in situ by x-ray photoelectron spectroscopy. Different thicknesses of the titanium overlayer were deposited on the SnO_2_ substrate kept at room temperature and at 200 °C. The spectral data showed significant chemical reactivity at the interface. The SnO_2_ was observed to become reduced to elemental tin. Complete reduction of the SnO_2_ was not observed even for the lowest thickness of the overlayer for both the types of deposition. The overlayer was observed to get oxidized to TiO_2_. The interface was also observed to consist of Ti-suboxide and unreacted Ti. Curve fitting was utilized to estimate the percentage composition of the constituents at the interface. Significant differences were observed for the two sets of the spectral data. The 200 °C data showed complete oxidation of the overlayer until a thickness of 0.9 nm. The Ti-suboxide showed a variation around 20% in the room temperature samples while an increasing trend was observed for the 200 °C samples. The complete oxidation of the titanium overlayer in the 200 °C samples has been interpreted as the thermal diffusion of the overlayer in the underlying SnO_2_ substrate. Thermodynamical considerations indicate the formation of TiO_2_ to be energetically favorable. The reduction of SnO_2_ to SnO in the presence of Ti is also possible. However, the experimental data do not demonstrate such a reduction. The nature and the amount of the constituents at the interface was observed to depend upon the substrate temperature. These constituents depend upon the annealing temperature, the annealing time, and the thickness of the overlayer. The interface can be subjected to a desired processing condition to obtain a device for a particular application. The present investigation also points to the need of exploring electrical characteristics of the interface against the processing conditions.

## Data Availability

Not applicable.
